# Pediatric Physiotherapeutic Approach for Guillain–Barre Syndrome Associated With Dengue Fever: A Case Report

**DOI:** 10.7759/cureus.46488

**Published:** 2023-10-04

**Authors:** Reva D Rajurkar, Deepali S Patil, Medhavi V Jagzape

**Affiliations:** 1 Physiotherapy, Ravi Nair Physiotherapy College, Datta Meghe Institute of Higher Education and Research, Wardha, IND

**Keywords:** flaccidity, strengthening, rehabilitation, pediatrics, faciliatory approach, dengue fever, autoimmune disease, guillain-barre syndrome

## Abstract

Guillain-Barre syndrome (GBS) is a peripheral nerve system (PNS) inflammatory disorder characterized by sudden, acute, symmetrical, generally ascending limb weakness with diminished or absent deep tendon reflexes, upper- and lower-extremity paresthesias, and sensory complaints. GBS is caused by an aberrant immunological response to an infection, which causes peripheral nerve damage. Dengue virus has been linked to a number of neurological diseases, including GBS. In the current case report, an eight-year-old child was taken to the hospital with dengue fever and lower limb paralysis. Physiotherapy methods focused on muscle strength and functional activity. The major goal of this case study was to assess functional tasks and enhance the patient's reaction to physical treatment. We find that the patient's response to strength and functional tasks was excellent in the early phases of recovery.

## Introduction

Guillain-Barre syndrome (GBS) is characterized by acute, symmetrical limb weakness that typically starts in the lower extremities and moves upward, accompanied by reduced or absent deep tendon reflexes and disordered tingling sensations in both the upper and lower limbs. This condition is of autoimmune origin, and individuals with GBS often experience dysfunction of cranial nerves, with the facial nerves and muscle groups being the most commonly affected [1,2]. It represents the rarest source of sudden paralysis, occurring at a worldwide rate of one to two cases per 100,000 individuals annually. GBS affects men more frequently than women, although it can strike anyone, and the likelihood of developing it rises with advancing age [3]. GBS manifests in several scientific subtypes, which encompass typical sensorimotor GBS, paraplegic GBS, sudden motion GBS, impulsive sensory GBS, Miller-Fisher syndrome (MFS), oropharyngeal-cervical-brachial (PCB) syndrome, bilateral facial palsy with tingling sensations, and brainstem GBS. Bickerstaff brainstem encephalitis represents a form of encephalitis within this spectrum [4]. Moreover, there are mutations [5]. The exact mechanism behind GBS remains unclear, but it seems to be initiated by the body's typical response to an illness, resulting in damage to the peripheral nerves. In a subset of GBS patients, elevated levels of serum antiganglioside antibodies are detected in the axolemma and other peripheral nerve elements [6,7]. Complement activation, macrophage invasion, and edema are frequent hallmarks of GBS-induced peripheral and neuromuscular events [6].

Arboviruses are the main cause of illnesses in humans globally [8]. Viruses, such as Zika virus (ZIKV), dengue, and chikungunya, cause devastating epidemics in tropical cities and are increasingly producing widespread outbreaks in the Western Hemisphere [9]. GBS and other neurological problems have been associated to dengue fever. Furthermore, in certain cases, these viral infections have been associated to neurological diseases [10]. Drowsiness, hyperreflexia, and Babinski reflex are unusual symptoms of the central nervous system. Muscle weakness, eye and vision problems, and trouble eating, speaking, or chewing are all possible symptoms. Acupuncture needles cause shivering in the hands and feet and serious pain that can hit at any time of day or night.

Coordination issues, unsteadiness, irregular heartbeat, or blood pressure levels are observed [11]. Due to the lack of considerably sensitive and specific sickness biomarkers, GBS is diagnosed based on medical history and examination, followed by further tests, such as cerebrospinal fluid (CSF) testing and electrodiagnostic studies [1]. GBS is often treated using a multimodal supportive care and immunotherapy approach. Intravenous immunoglobulin (IVIG) and plasma exchange have both been proven to be successful GBS therapies [12]. Creating a rehabilitation plan with a therapy physician, physical therapist, or occupational therapist is an important first step toward recovery. The purpose of the program is to reduce handicap in the early stages of recovery before motor, sensory, and fitness levels restore to pre-illness levels. Exercise, stationary cycling, walking, and power training have all been shown to improve GBS patients' fitness, walking ability, and independence in everyday sports. However, exercise intensity must be carefully monitored because overexertion can lead to fatigue [13].

## Case presentation

Patient information

An eight-year-old male child was admitted to Acharya Vinoba Bhave Rural Hospital, Sawangi (Meghe), Wardha, India. On February 5, 2023, he complained of fever, and on February 8, 2023, he was diagnosed with dengue fever. He also had history of dengue two months before. He was on medications, such as oral rehydration therapy and acetaminophen, for fever and recovered completely from dengue. The patient complained of weakness in the lower limb and body pain. The weakness onset was gradual. He had weakness in the lower back and had difficulty while sitting. He faced difficulty in performing daily living activities. He was unable to stand and has difficulty in maintaining static and dynamic balance. He was referred to the neurology department and diagnosed with GBS.

Clinical findings on day 1

Clinical findings revealed loss of power in both lower limbs. Table 1 shows the manual muscle grading of the lower limb by the Medical Research Council (MRC). CSF findings showed elevation in the protein level. Lower weakness was more profound compared to the bilateral upper limb. No visual deficits, no cognitive changes, and no sphincter involvement was seen. The patient had early fatigue and body pain and was not able to experience daily living activities, such as walking, standing, and sitting. The touch, pain, and temperature sensations in the lower limb were found to be diminished, whereas in the upper limb, it was found to be intact. Manual muscle power testing revealed grade 2 power in the bilateral lower limb, whereas the upper limb showed muscle power of grade 3. Deep tendon reflexes revealed hyperreflexia. Muscle tightness revealed tightness in the hamstring and tibialis anterior in the bilateral lower limb.

**Table 1:** Manual muscle test (MMT) grades of the lower limb

Muscle	Right	Left
	Hip	
Flexion	2	2
Extension	2	2
Abduction	2	2
Adduction	2	2
	Knee	
Flexion	3	3
Extension	3	3
	Ankle	
Dorsiflexion	2	2
Plantarflexion	2	2

Clinical diagnosis

The patient was investigated for several blood tests, such as CBC, peripheral smear, creatine kinase (CK), CK-MB, C-reactive protein (CRP), kidney function test (KFT), and liver function test (LFT). Lumbar puncture was also done for CSF findings. The lumbar puncture and clinical findings allowed the final diagnosis of GBS.

**Figure 1:**
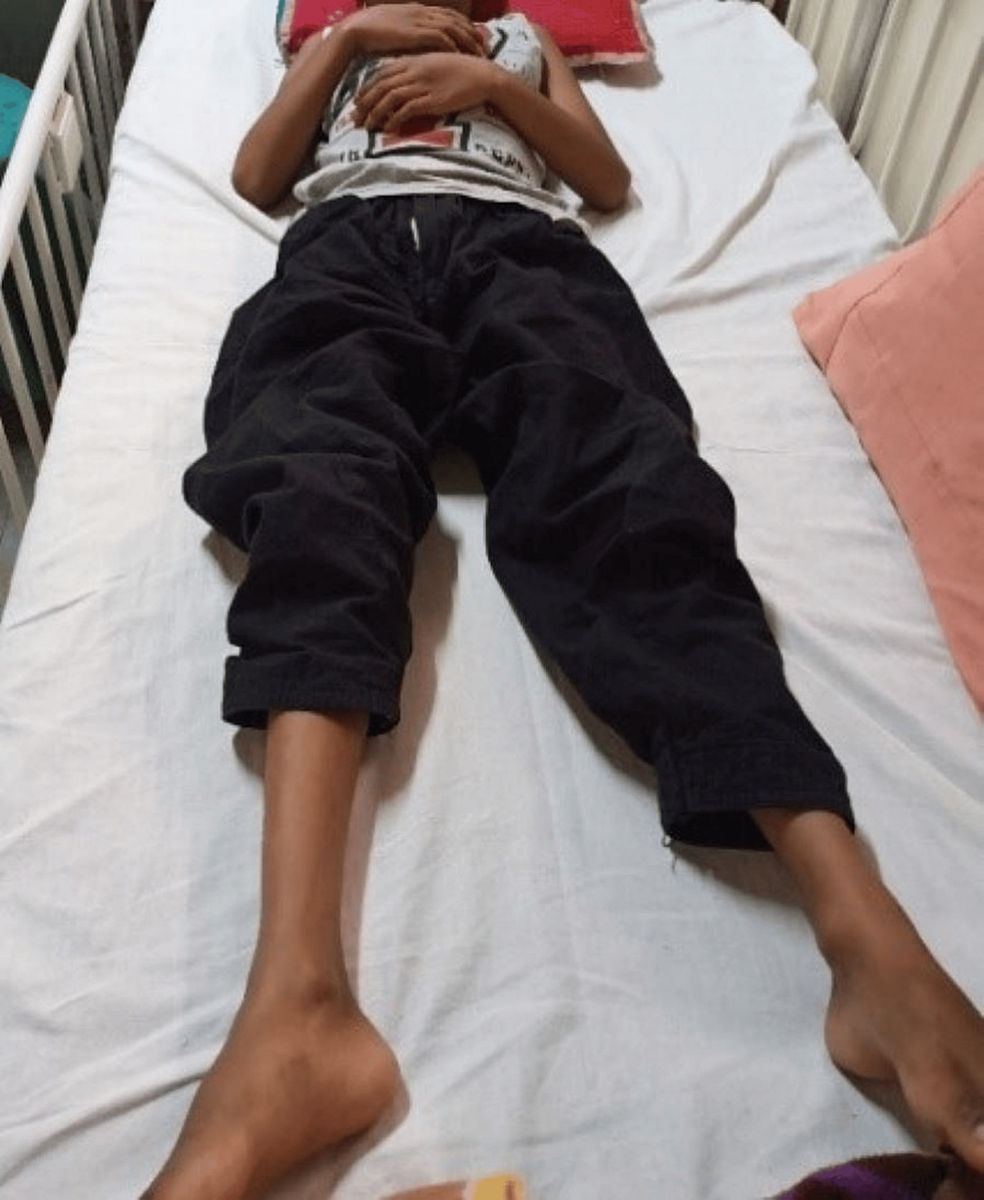
Patient lying in supine position

Figure 1 shows the patient posture at clinical day 1, in supine lying, with hip and knee in neutral.

Therapeutic intervention

Physical rehabilitation primarily aimed at early intervention to avoid secondary complications, such as joint contractures, deformities, pressure sores, and other chest complications. Parents and caregivers were instructed to give proper positioning and constant change in positioning every two hours. Rehabilitation program was given twice a day, which included breathing exercises and passive and active assisted range of motion exercises to all the joints of both extremities. All the activities were performed at a pace to prevent fatigue and according to the patient’s comfort.

Table 2 shows the treatment details according to the problem list.

**Table 2:** Treatment details

Problem	Cause	Therapeutic goals	Treatment
Reduced chest compliance	Prolonged immobility	To improve vital capacity	Deep breathing exercises, glossopharyngeal breathing exercises, teaching coughing techniques
Prevention of pressure sores	Prolonged immobility	To maintain proper skin care	Positioning using pillows. Position: change every two hours. Teaching pressure relieving maneuver (to be done also while sitting on the wheelchair)
Reduced range of motion	Due to the injury to the spinal cord	Maintain joint integrity and available range of motion	Passive range of motion exercises. Positioning In order to maintain alignment for functional activities. Selective stretching to improve function.
Reduced strength	Prolonged Immobility	Maintain available muscle strength	Strengthening exercises for the upper extremities
Patient and caregiver education			Explain the home exercise programme in easy language. Explain all warning signs, do’s and don’ts.

Clinical findings on day 21

In the 21-day post-intervention, there was a significant improvement in the patient's sensory and motor status. Manual muscle test (MMT) revealed muscle power 3 in the bilateral upper limb and 4 in the bilateral lower limb. Moreover, all the superficial sensations were found to be in the bilateral upper and lower limbs. Now, the patient could sit bedside with support, which showed improvement in balance. Moreover, the patient could perform daily living activities with minimal assistance.

**Figure 2:**
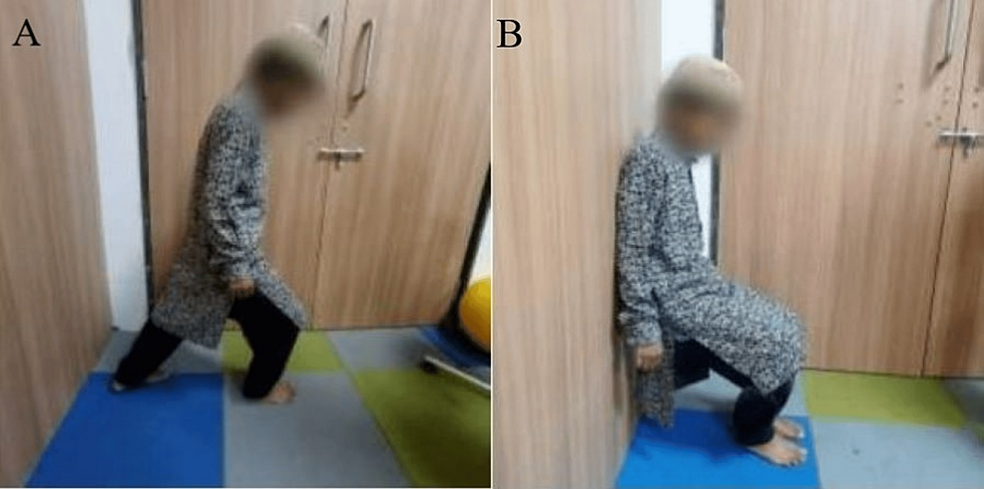
Postural stability and balance exercise: A postural stability; B balance exercise

**Figure 3:**
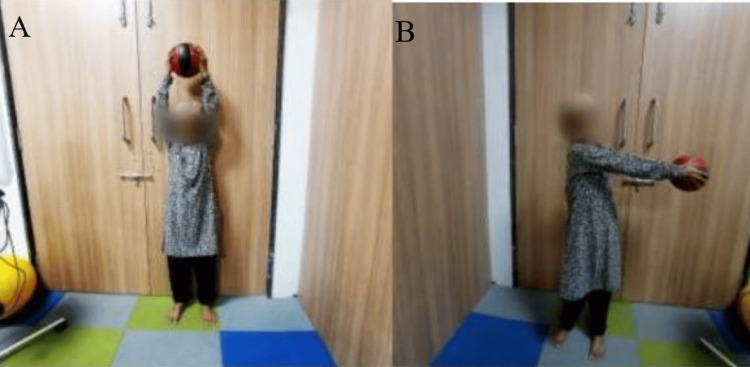
Strengthening exercise using a Thera ball: A upper limb flexor strengthening; B upper limb abductor strengthening

**Figure 4:**
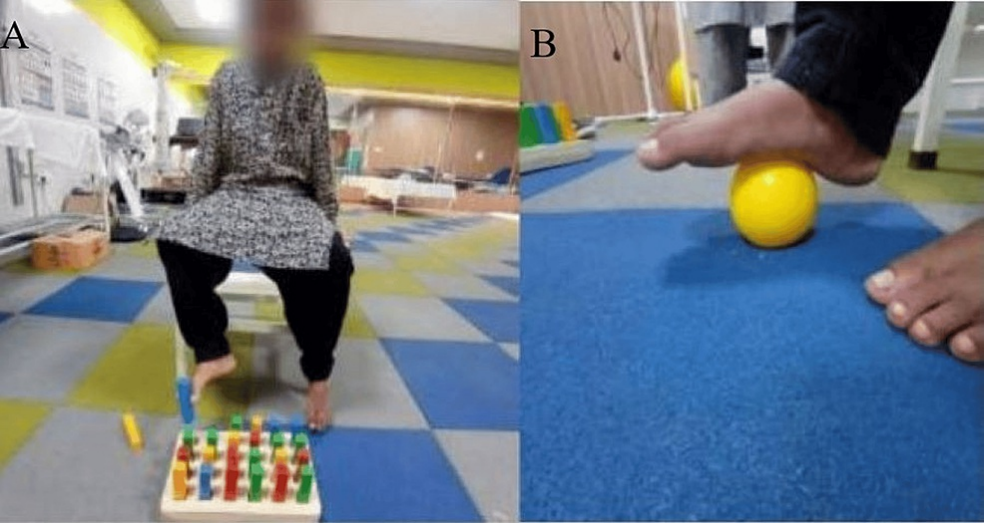
A grasping objects with the toes; B rolling ball by the foot

Figure 2 shows the patient practicing an exercise for postural stability and balance. Figure 3 shows the patient practicing a strengthening exercise using a Thera ball. Figure 4A shows a grasping exercise with the toes, and Figure 4B shows a rolling exercise with the foot.

## Discussion

GBS is a rapidly progressing autoimmune disease that damages the peripheral nerve system (PNS). GBS is distinguished by progressive muscle fatigue, absent or reduced reflexes in the affected limbs, pain, and occasionally autonomic dysfunction due to demyelination of the myelin sheath. In the present case, the healing process was a critical component of the rehabilitation program because the patient came from a rural setting and was uneducated; adequate patient education was required and result-oriented treatment was required to meet his expectations. The primary areas of concern for physiotherapy therapies are initial acute care, where life must be saved by focusing more on the chest, impairments of strength, impaired coordination, and functional limitations. However, researchers estimate that these deficits account for roughly 40-50% of life quality reduction expectations. IVIG is delivered to a patient with GBS. The immunological system (the physique's natural defences) produces toxins that attack the nerves, resulting in GBS. IVIG is a medicine that is made from antibodies generated from healthy donated blood. These are given to assist prevent your nerves from getting harmed by harmful antibodies. IVIG is an immunoglobulin that is inserted directly into a vein [14]. After medical management, the patient reported to the physiotherapy department for rehabilitation. He was unable to walk on his own. We planned physiotherapy in order to increase his strength and decrease spasticity and for gait training, among others.

Our study had some limitations, including the fact that it was a single-center, one-case-only, and hospital-based observational study; generalizing these findings should be done with caution. More study in a bigger series with a longer follow-up is required [15].

## Conclusions

An accurate treatment plan focusing on strength and functional tasks and educating the patient's parents on the importance of post-discharge care was extremely beneficial to the patient. The patient's strength and functional behaviors improved initially, but he still required follow-up treatments. Although the patient was unable to do his regular activities in the current study, he learned the importance of physiotherapy and the challenges he would face if he did not receive treatment.
